# The Role of Fundus Autofluorescence Imaging in the Study of the Course of Posterior Uveitis Disorders

**DOI:** 10.1155/2015/247469

**Published:** 2015-01-28

**Authors:** Panagiotis Malamos, Panos Masaoutis, Ilias Georgalas, Stelios Maselos, Konstantinos Andrianopoulos, Chryssanthi Koutsandrea, Nikos N. Markomichelakis

**Affiliations:** ^1^Department of Ophthalmology, Ocular Immunology and Inflammation Service, “G. Gennimatas” General Hospital of Athens, Athens, Greece; ^2^1st Department of Ophthalmology, “G. Gennimatas” General Hospital of Athens, University of Athens, 59 Chrysnathemon Street, Psychiko, 15452 Athens, Greece; ^3^Institute of Ocular Inflammation and Pathology of the Eye, Athens, Greece

## Abstract

*Background.* To evaluate the correlation of fundus autofluorescence (FAF) with indocyanine green angiography (ICGA) in patients with various posterior uveitis disorders. *Methods*. Interventional case series including 23 eyes of 15 patients with diagnosis of a specific type of retinochoroiditis, such as acute posterior multifocal placoid pigment epitheliopathy (APMPPE), serpiginous-like choroiditis, multifocal choroiditis (MFC), Harada disease, and syphilitic retinochoroiditis. Also, some cases with undefined retinochoroiditis were included. FAF and ICGA were performed and correlated at baseline and during follow-up after treatment. *Results*. In ICGA, early hypofluorescence was found to be the hallmark of acute choroidal inflammation, resolving in later stages and remaining in the late phase in areas with retinal pigment epithelium (RPE) damage. Poorly defined hyperautofluorescent areas correlated with acute choroidal lesions. Hypoautofluorescent delineation suggested the initiation of RPE healing processes, correlating well with the late phase of ICGA and delineating the RPE damage. Early hyperautofluorescence with late hypofluorescence in ICGA indicated the presence of primary RPE involvement. *Conclusion*. FAF contributes to the interpretation of RPE disease and may be a useful tool for the follow-up of progressive inflammatory disorders. Comparative evaluation of FAF and ICGA allows a characterization of the sequence of inflammatory events and the level of tissue affected.

## 1. Introduction

Posterior uveitis is a general classification term, which includes various inflammatory disorders, affecting primarily the retina, the choroid, or both. In cases of multilevel involvement, discrimination of the initially or exclusively affected tissue allows a more precise classification into retinitis, choroiditis, or retinochoroiditis. Diagnosis and follow-up of posterior uveitis patients have evolved significantly with the advances in imaging technology, such as indocyanine green angiography (ICGA) and fundus autofluorescence (FAF). These imaging tools are complementary to the standard evaluation with fundoscopy and fluorescein angiography (FA) [1, 2].

Retinal inflammatory diseases, manifesting mainly as vasculitis or necrotic retinitis, such as Behcet uveitis, ocular toxoplasmosis, and herpes-associated acute retinal necrosis, can be easily suspected or even detected during fundoscopic examination. In cases of suspected retinal vasculitis or secondary induced ischemia, FA is a very useful tool for the clinical diagnosis [3–7]. However, inflammation of the choroid and retinal pigment epithelium (RPE) complex often remains occult on thorough fundoscopic examination and FA imaging. This is because the initial site of inflammatory insult is partially obscured by the overlying tissues of the neurosensory retina and RPE. Former “white dot syndromes” (WDS) and choroidal stroma inflammatory disorders are typical examples of this. In such cases, ICGA is more appropriate to detect the full extent of choroidal inflammation and consequently contributes significantly to an adequate differential diagnosis and follow-up of the disease [8–11].

In the last years, noninvasive FAF imaging has been introduced into the ophthalmological clinical practice, providing for the first time a qualitative measure of the status of RPE layer in terms of function and structure [12, 13]. This has been shown to be especially useful for the evaluation of various retinal disorders [14–16]. As a result, there has been an increased interest in the role of FAF in the assessment and follow-up of inflammatory diseases of retina and RPE-choroid complex [17–23].

In an attempt to optimize diagnostic procedures, there is a need to identify the most informative and least invasive clinical protocol for the evaluation of posterior uveitis. To our knowledge, there is no published study to date exploring the correlation between ICGA and FAF in patients with different forms of posterior uveitis. This analysis may provide a better understanding of the diagnostic potential of noninvasive FAF imaging. In the current study, our aim was to investigate if the combined-mode imaging (ICGA+FAF) could provide useful additional information contributing to the diagnosis, follow-up, and treatment optimization in posterior uveitis.

## 2. Methods

This study is an interventional case series conducted at the Department of Ocular Immunology and Inflammation of “G. Gennimatas” General Hospital in Athens, Greece. Informed consent was obtained from all participants. All procedures were done in adherence to the tenets of Declaration of Helsinki. The study was approved by the institutional ethics committee.

All patients included in the study either were new referrals, with symptoms or signs of posterior segment inflammation, or were already diagnosed with uveitis, with a follow-up performed in our department. At baseline, all patients had a complete ophthalmological examination, including biomicroscopy and fundoscopy. In addition, FAF exam, ICGA, and FA were performed. A standardized ICGA protocol for posterior uveitis was followed [9], using the HRA 2 Spectralis Heidelberg digital angiography system (Heidelberg, Germany). Besides these tests, the outer/inner retinal layers were evaluated by means of the RTVue spectral domain optical coherence tomography (SD-OCT) system (Optovue Inc., Fremont, USA).

Pharmacological treatment was prescribed when necessary. During the follow-up, FAF and ICGA examinations were repeated when the signs of acute inflammation subsided as a result of treatment or due to a self-limiting clinical course. Direct comparison was performed between baseline and follow-up images obtained with both FAF and ICGA to investigate the presence of clinically relevant correlations of the results obtained with the two imaging technologies. Likewise, the level of affected tissue at baseline and during follow-up was evaluated. The clinical course of each disease and its anatomic and functional impact on the eye structures were also documented.

## 3. Results

23 eyes of 15 patients with various types of posterior uveitis were included. Specific diagnosis was done based on the results of FAF, ICGA, OCT, and FA imaging in most of cases (Table 1). All cases were classified according to the main pathophysiologic mechanisms as WDS, currently named inflammatory choriocapillaropathies [24, 25], or stromal choroiditis [26, 27]. Six eyes of 4 patients had an undefined diagnosis despite thorough clinical, imaging, and laboratory assessment. In these cases, the sequence and extent of involvement of the various chorioretinal structures could be identified. A combined evaluation with FAF and ICGA was performed for each clinical entity.

### 3.1. Acute Posterior Multifocal Placoid Pigment Epitheliopathy (APMPPE)

**Figure 1:**
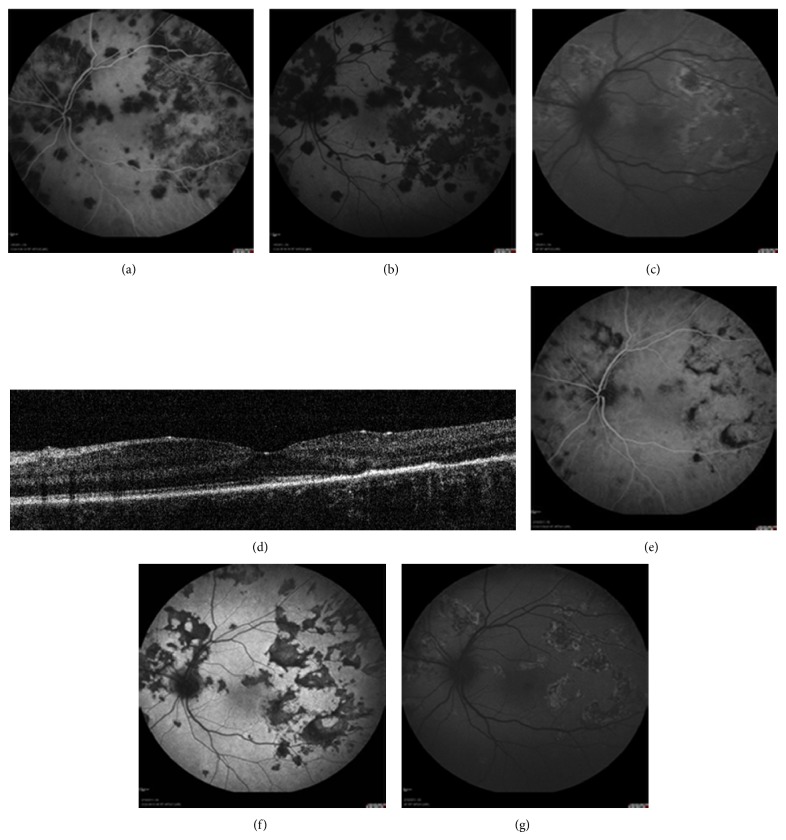
Acute posterior multifocal placoid pigment epitheliopathy (APMPPE). ICGA early phase (a), ICGA late phase (b), FAF (c) and OCT (d) at baseline prior to treatment. ICGA early phase (e), ICGA late phase (f) and FAF (g) at follow-up after treatment. Concordance between late phase ICGA (f) and FAF (g) after treatment is the rule.

Discrete contiguous hypofluorescent areas of the posterior pole are characteristic of acute disease in ICGA. In our series, two different angiographic patterns were observed in different areas. In the first one, profoundly dark hypofluorescent patches appeared in the early phase of the ICGA exam and persisted throughout the complete sequence of the exam. In the other pattern, hypofluorescent areas appeared mainly in the late phase (Figures 1(a) and 1(b)).

FAF imaging revealed well-defined lesions with stippled autofluorescence in the core and hyperautofluorescent (hyper-AF) borders, which were partially surrounded by hypoautofluorescent (hypo-AF) halos (Figure 1(c)). These FAF lesions corresponded mainly with areas of late hypofluorescence in ICGA. Poorly defined and barely visible hyper-AF lesions were found in those areas showing hypofluorescence in the early phase of ICGA. SD-OCT in the acute stage revealed a disruption of the photoreceptor layer as well as focal hyperreflective lesions in the inner retina (Figure 1(d)).

In the convalescent stage, the early phase hypofluorescent areas (seen acutely) seemed to have faded away in ICGA. On the other hand, late phase hypofluorescence remained unchanged. Correlation between FAF and late phase ICGA seemed to be much stronger in the convalescent compared to the acute stage in all cases. (Figures 1(f) and 1(g)).

### 3.2. Tubercular Serpiginous-Like Choroiditis

**Figure 2:**
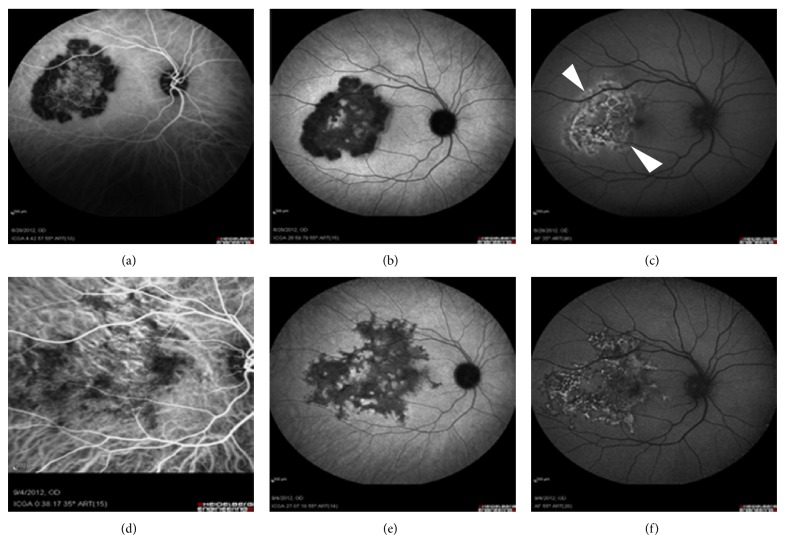
Tuberculosis associated serpiginous-like choroiditis. In the acute phase, a hypofluorescent rim in ICGA (a, b) delineates the edge of the progressive lesion, appearing in FAF (c) as poorly defined hyperautofluorescent margins (arrowheads). Lesion has experienced an expansion peripherally (d, e) and is surrounded by a hypo-AF rim (f) after treatment.

Four eyes of 4 patients with serpiginous-like choroiditis, in the presence of a positive Mantoux and Quantiferon gold test, were included. In the acute stage, ICGA revealed profoundly dark hypofluorescent areas during the complete sequence that surrounded areas of hyperfluorescence in a serpiginous or centrifugal pattern (Figures 2(a) and 2(b)). FAF images showed lesions with a lacey hyper-AF pattern in the acute stage. These hyper-AF areas were partially surrounded by a well-defined iso- or hypo-AF band with a thin poorly defined hyper-AF border (Figure 2(c)).

In the convalescent stage, the majority of early-acute hypofluorescent areas became invisible in ICGA after treatment application. There was an agreement between the late phase of ICGA and FAF images. Furthermore, a clearly defined hypo-AF rim delineated the periphery of the whole AF lesions, which seemed to have undergone a peripheral expansion compared to baseline (Figures 2(d), 2(e), and 2(f)).

### 3.3. Multifocal Choroiditis Panuveitis (MCP)

**Figure 3:**
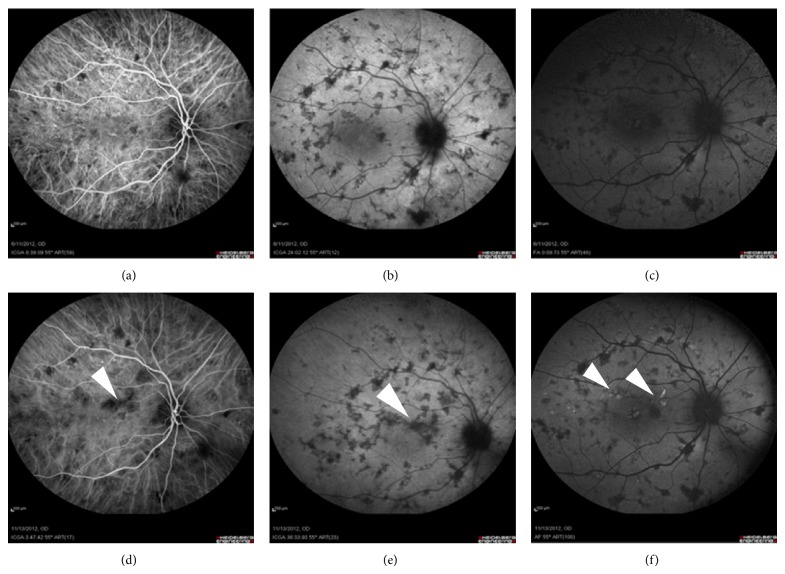
Multifocal choroiditis (MFC). Initially, ICGA and FAF show numerous small hypofluorescent spots randomly scattered in the posterior pole (a, b, c). In the follow-up, the onset of new hypofluorescent spots in ICGA (d, e) correlates well with the presence of hyperautofluorescent spots (f) in areas of previously normal background.

In MFC, ICGA revealed multiple hypofluorescent spots which became more obvious and numerous in the late phase. These findings in the late phase of ICGA correlated well with hypo-AF lesions on FAF (Figures 3(a), 3(b), and 3(c)). On further follow-up, new hyper-AF spots (Figure 3(f), arrowhead) seem to correspond partially with hypofluorescent defects in ICGA (Figures 3(d), 3(e), and 3(f)).

### 3.4. Acute Syphilitic Posterior Placoid Chorioretinitis (ASPPC)

**Figure 4:**
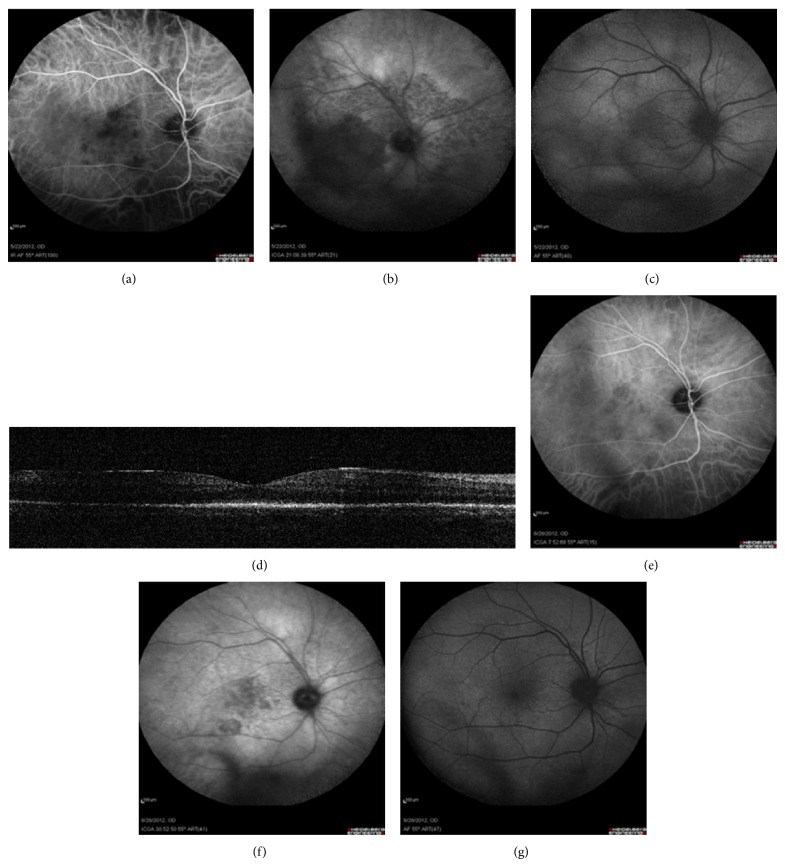
Acute syphilitic posterior placoid chorioretinopathy (ASPPC). Early phase ICGA shows minimal choroidal signs (a). Diffuse late phase mottled hypofluorescence in the posterior pole (b) corresponds to hyperautofluorescence in FAF (c). An extensive disruption of IS/OS layer is evident on OCT images (Figure 4(d)). There was a resolution of acute findings after treatment (e, f, g).

In the active/acute stage of the disease, ICGA revealed the presence of small hypofluorescent areas in the early phase that changed to a diffuse mottled hypofluorescence in the late phase. The latter largely corresponded with the diffuse hyper-AF area in the posterior pole as seen on FAF. SD-OCT showed a disruption of RPE and outer retina (Figures 4(a), 4(b), 4(c), and 4(d)).

After finishing the appropriate treatment, a resolution of hypofluorescence in ICGA and hyper-AF in FAF was observed that was accompanied with the disappearance of inflammatory signs and gains in visual acuity (Figures 4(e), 4(f), and 4(g)).

### 3.5. Harada Disease

**Figure 5:**
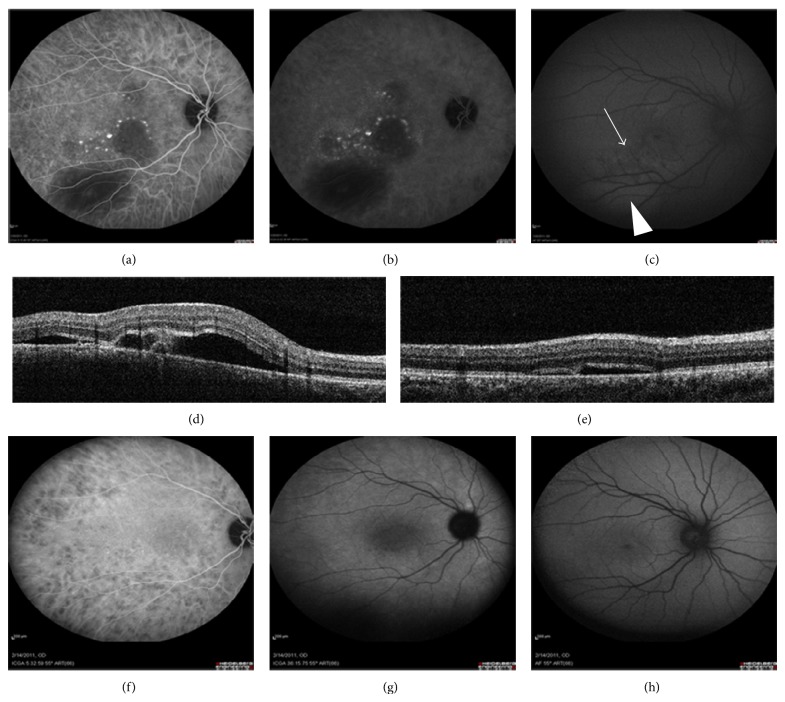
Acute Vogt-Koyanagi-Harada disease (VKH). Initially, hypofluorescent patches in ICGA surrounded by hyperfluorescent spots (a, b) correspond to areas of exudative detachments. They appear faintly hyperautofluorescent in FAF (Figure 5(c) arrowhead). OCT shows a multilobe neurosensory detachment (Figure 5(d)). There was a significant decrease of inflammatory signs after treatment (e, f, g, h).

In the acute stage of the disease, ICGA showed hypofluorescent dots in the early phase that were converted into hyperfluorescent spots in the mid and late phase. Large circular or oval areas of hypofluorescence (representing areas of neurosensory retinal detachment) were found to be surrounded by hotspots. (Figures 5(a) and 5(b)). FAF images showed poorly defined areas of hyper-AF that were partially surrounded by mixed faint hyper- and hypo-AF dots. (Figure 5(c), arrow and arrowhead). SD-OCT showed a characteristic pattern of multilobular exudative detachment of the neurosensory retina that resolved after treatment (Figures 5(d) and 5(e)). In the convalescent stage, a nearly complete resolution of FAF lesions was observed, whereas small hypofluorescent dots remained in ICGA in the mid phase (Figures 5(f), 5(g), and 5(h)).

### 3.6. Undefined Cases

**Figure 6:**
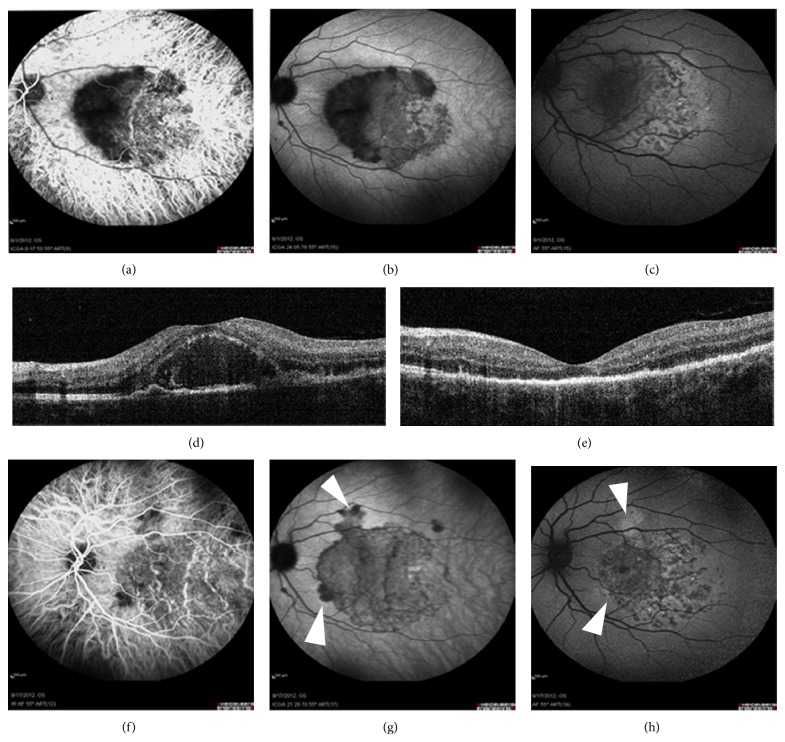
Full-thickness inflammation of retina-choroid of undefined origin. Initially, ICGA shows choroidal ischemia as central hypofluorescence (a, b). FAF shows a hyperautofluorescent plaque temporal to ischemia (c). In OCT, serous detachment (d) decreases after treatment (e). Despite reperfusion of ischemia (f), new satellite lesions (arrowheads) appear in ICGA and FAF (g, h), indicating a peripheral progression of the disease.

Two cases were classified as full-thickness inflammation of choroid and retina in the macular area. Their common characteristic was a significant macular hypofluorescence in ICGA that was compatible with choroidal ischemia (Figures 6(a) and 6(b)). FAF showed diffuse mottled, plaque-like hyper-AF (Figure 6(c)), suggesting a potential RPE inflammatory involvement in the acute stage. A corresponding outer segment disruption and neurosensory macular detachment was evident on OCT (Figure 6(d)). In the healing stage, the reperfusion of the choroidal ischemia in ICGA was combined with a resolution of the macular detachment on OCT examination (Figure 6(e)). FAF imaging showed a mixed pattern of altered AF and provided an evidence of progression in the periphery (Figure 6(h) arrowheads), that was confirmed with ICGA (Figures 6(f) and 6(g) arrowheads).

## 4. Discussion

As previously commented, there are specific diagnostic tools available in clinical practice, to evaluate the inflammatory disease of the posterior segment of the eye. These tools include standard methods, such as FA and ICGA, OCT, and the novel technology FAF, which is being increasingly utilized as a noninvasive imaging procedure in the field of uveitis. FAF imaging is based on the special fluorescence properties of various molecules found in ocular fundus collectively called “fluorophores.” These molecules can be excited by electromagnetic energy (light) to a higher energy level and then return to their previous state by emitting light of higher wavelength. The major source of autofluorescence in human fundus is lipofuscin. This term describes a heterogeneous group of fluorophores which are found in the RPE cells and are the end product of incomplete lysosomal digestion of phagocytosed photoreceptor outer segments. Increased fundus autofluorescence is observed with aging but also in pathologic conditions whereas decreased autofluorescence is noted in RPE atrophy and/or due to signal attenuation from other pigments [28, 29]. In the setting of chorioretinal inflammation, the affected RPE monolayer shows increased metabolic activity. This metabolic strain is thought to generate, through alternative metabolic pathways, an excess of substances with potential autofluorescence properties [30–32]. In such setting hyper-AF could be used as a surrogate marker of active inflammation at the RPE level. In this study, we evaluated the correlation between ICGA and FAF in patients with different forms of posterior uveitis and if the combination of both imaging technologies could provide additional clinically relevant information, contributing to the diagnosis and treatment optimization in these types of patients.

Most of cases included in our series were classified under the general term WDS that represents a wide spectrum of diseases, such as APMPEE, serpiginous choroiditis, and MCP. These conditions, despite the variability of the clinical manifestations, may share common pathophysiologic mechanisms. Recently, choriocapillaris inflammation and secondary ischemia have been proposed as the initial events in WDS. However, it remains controversial if the inflammatory sequence originates in other neighboring structures such as the RPE, as was initially hypothesized [31]. A comparative evaluation of FAF and ICGA outcomes may possibly shed more light on such a distinction and facilitate our diagnostic approach.

In our APMPPE cases, ICGA provided better imaging of the disturbances occurring in the choroidal circulation. Acute inflammation of choriocapillaris layer with secondary ischemia is the initial event followed by a reperfusion state at a later, convalescent stage. ICGA allowed us to obtain a precise mapping of these stages that coexisted in discrete geographic areas at the posterior pole, in the form of multiple, polymorphous, and asynchronous patches. The analysis of the correlation between ICGA and FAF allowed us to obtain an accurate evaluation of the inflammatory impact occurring at the RPE level, in terms of time and extent. Well-defined FAF findings were apparent only in areas of reperfused choriocapillaris, suggesting an established RPE damage. In contrast, minimal, ill-defined, hyper-AF spots with fluffy margins correlated with areas of acute choriocapillaris inflammation, which imply that there was not remarkable initial RPE involvement at the time of primary disturbance in the choroidal circulation.

During the convalescent stage, lesions in the late phase of ICGA and FAF presented a remarkable similarity in size and shape. According to these outcomes, both FAF and late phase ICGA seemed to delineate the whole area of functionally impaired RPE, remaining after the resolution of acute inflammation. FAF imaging had the additional advantage of providing more information on the evolution and age of lesions. Nevertheless, OCT imaging, used in a complementary fashion, can further detect functional and structural alterations of the outer retina induced by the ischemia of underlying choriocapillaris. Overall, the area of initial choriocapillaris damage seemed to be much more extensive than the final RPE involvement, considering that not all acute lesions observed in ICGA were transformed into an abnormal FAF signal in the subacute or convalescent stage. Obviously, the size of the acutely ischemic areas at the choriocapillaris level may be the critical factor for the conversion of ischemia into an established RPE damage. Relatively small ischemic lesions were found to fade away in the follow-up, not affecting the overlying RPE (Figures 1(a) and 1(g)).

Acute choriocapillaris inflammation may be the initial site of the pathological process in tubercular serpiginous-like choroiditis as well. In our series, FAF clearly identified the area of established RPE damage that corresponded with the reperfused choriocapillaris layer underneath. Likewise, hyper-AF margins were shown to be an indicator of an active underlying choroidal inflammation. Similar evidence has been recently reported by Gupta et al. in a series of serpiginous-like cases with associated tubercular infection [33]. In the presence of poorly defined hyper-AF margins with no hypo-AF delineation (Figure 2(c), arrowheads), the presence of an active choroidal disease should be suspected and ICGA should be performed in order to investigate its real extent. Expansion of the initial RPE lesion in FAF images may be expected in these cases (Figures 2(e) and 2(f)). On the other hand, some FAF lesions were found to be completely surrounded by hypo-AF margins, suggesting the initiation of a healing process at the RPE and the inactivity of the choroidal inflammation, confirmed by ICGA imaging. In such cases, no peripheral expansion of the lesion should be expected. In contrast to APMMPE, final RPE damage in serpiginous-like choroiditis due to tubercular infection, evaluated by means of follow-up FAF mapping, seemed to extend beyond the margins of the initial inner-choroidal involvement outlined in the baseline ICGA.

Regarding MCP, the analysis of the correlation between the two imaging modalities, ICGA and FAF, did not show any significant discrepancy in the findings at the inner-choroid and RPE level, as observed in the acute stage of the previous entities evaluated. In this condition, FAF was found to provide a better and more precise imaging of the RPE involvement than ICGA or fundoscopy in the form of hypo-AF spots scattered in the posterior pole. As choroidal structures seemed to be minimally affected, ICGA did not reveal significant findings in the early phase. ICGA was demonstrated to be inferior to FAF in the imaging of defects, even in its late phase. Indeed, the superiority of FAF over ICGA was observed to be even more significant in cases with exacerbations. In these cases, new hyper-AF spots in sites of previously normal background AF were confirmed to be indicative of a progression of the disease (Figure 3(f)). According to all these findings, it was obvious that RPE defects in MCP as visualized in FAF and late phase ICGA exceeded by far choroidal changes and had a permanent and not resolving course during follow-up. This contrasts with the outcomes obtained in APMPPE, showing a prominent choroidal involvement that did not always reach the adjacent RPE. It is possible that in MCP, as suggested by some authors, RPE is affected before or simultaneously with the choroid, but to a larger extent. This may explain the presence of a more significant RPE damage compared to changes in the choroid [18, 34]. More studies on the pathogenic mechanism of MCP are still necessary to confirm all these hypotheses.

In ASPPC, the primary inflammation in the posterior pole has been postulated to be of infectious origin at the RPE/outer retina with a placoid pattern [35]. In our series, ICGA showed minimal involvement of the choroid in early phase, with a profound hypofluorescence in late phase that constitutes a marker of the diffuse functional impairment of RPE. This may be attributable to atrophy, healing, or an acute inflammatory event at the RPE level. FAF was observed to provide this missing information, suggesting that a primary acute RPE inflammation in the form of diffuse increase of AF signal in the posterior pole was present. SD-OCT ruled out the presence of subretinal fluid, which would had provided similar FAF findings and revealed the presence of a disruption of the outer retina at the level of inner/outer segment junction of photoreceptors. This was consistent with the significant visual loss that was present despite the few angiographic and clinically visible fundus signs. The resolution of acute FAF and ICGA findings after treatment correlated well with the level of restoration of the visual function achieved.

In the group of disorders classified as stromal choroiditis, a granulomatous inflammation within the choroidal stroma targeting the choroidal melanocytes has been proposed to be the underlying pathophysiologic mechanism [26]. Harada disease is discriminated from the other disorders of this category in the presence of exudative detachments at the acute stage. In this condition, ICGA was confirmed to be the procedure of choice in the imaging of the granulomatous choroidal disease, whereas SD-OCT was shown to provide the characteristic image of multilobular detachment of the neurosensory retina [36]. The role of FAF in this disorder was restricted to the detection of exudative detachments as geographic patches of increased signal, encircled by faintly visible, poorly defined hyper-AF dots. These dots may be an indicator of focal ischemic RPE lesions through which subretinal fluid may reach the subretinal space, inducing a neurosensory detachment. Subretinal space has been already established as potential source of increased AF signal, attributable to the impaired metabolism and phagocytosis of photoreceptors' outer segment tips, as a result of the poor apposition between RPE and neurosensory retina [37, 38].

In conclusion, the evaluation of the correlation of FAF and ICGA can provide clinically relevant and complementary information about the timing and extent of RPE involvement compared to inner-choroid structures in disorders of the choroid-RPE complex.  Table 2 summarizes in which specific cases the information provided by both imaging technologies seems to be especially useful, according to the outcomes of our limited series. FAF should not be considered as a substitute of ICGA in such inflammatory conditions but rather as a noninvasive complementary diagnostic and prognostic tool. This combination of imaging techniques allows the clinician to achieve a more detailed characterization of specific inflammatory clinical entities affecting the posterior pole. Once the diagnosis is clear, noninvasive FAF imaging could be used as a single follow-up tool, in cases of serpiginous-like choroiditis and MCP. In APMPPE, the inner-choroidal layer is affected first in the form of ischemic choriocapillaris inflammation and to larger extent than its corresponding impact on RPE. The limited, end-stage impact on RPE may be the basis of the benign course of this self-limited disease, with relatively favorable prognosis for the visual function. This should be confirmed in future studies. In serpiginous-like choroiditis (in the presence of active tuberculosis), the inflammatory sequence seems to also start at the choriocapillaris level, but the final impact on RPE is more severe. The boundaries of the final RPE damage exceed the area of the initial inner-choroidal lesion. This increased extent of RPE damage may be the reason for a more aggressive course of this disease, especially in cases of delayed diagnosis and inappropriate treatment, with less favorable prognosis for visual rehabilitation than other WDS. In contrast, in MCP and ASPPC, RPE seems to be the primary and predominant site of the inflammatory attack, even before affecting the choroid. Furthermore, relevant screening of the inflammatory activity can be obtained by observing the margins of preexisting lesions in serpiginous-like choroiditis or the development of new lesions in MCP (Table 2). All these findings must be confirmed in future studies with larger samples sizes and longer follow-up.

## Figures and Tables

**Table 1:** Distribution of patients included in the study according to the specific entity of posterior segment inflammation.

Diagnosis	Patients	Eyes
APMPPE	3	5

TB-serpiginous-like	4	4

MCP	2	4

ASPPC	1	2

Harada	1	2

Undefined		
Affected Tissue level		
RPE	1	2
Inner-choroid	1	2
Full-thickness	2	2

APMPPE: acute posterior multifocal placoid pigment epitheliopathy; TB: tubercular; MCP: multifocal choroiditis panuveitis; RPE: retinal pigment epithelium.

**Table 2:** Classification of diseases according to the affected structures.

		Disease				
		White dot syndromes				Stromal choroiditis
		APMPPE	Serpiginous-like choroiditis	MCP	ASPPC	Harada
Affected tissue (timing-extent)	Choriocapillaris	Primary **Predominant**	Primary	Secondary	Minimal	—
	Choroidal stroma	Secondary (nonperfusion)	Secondary (nonperfusion)	Secondary	Minimal	Primary **Predominant**
	RPE	Secondary	Secondary **Predominant**	Primary **Predominant**	Primary **Predominant**	Minimal
	Outer/inner retina	Secondary (outer)	Secondary (outer)	Secondary (outer)	Secondary (outer)	Secondary (outer)

Proposed imaging	Initial	FAF-ICGA	FAF-ICGA	FAF-ICGA	FAF-ICGA-OCT	OCT + ICGA
	Follow-up	FAF-ICGA- (OCT)	**FAF**- (OCT)	**FAF**- (OCT)	**FAF**-OCT	OCT + ICGA

APMPEE: acute posterior multifocal placoid pigment epitheliopathy; MCP: multifocal choroiditis panuveitis; ASPPC: acute syphilitic posterior placoid chorioretinitis; RPE: retinal pigment epithelium; FAF: fundus autofluorescence; ICGA: indocyanine green angiography; and OCT: optical coherence tomography. Primary: the tissue/structure affected first; secondary: tissue affected after the primary attack; **predominant:** the tissue affected in most extent; OCT: complementary imaging for evaluation of photoreceptors layer; minimal: tissue affected in less extent.
